# Modulation of mu rhythm desynchronization during motor imagery by transcranial direct current stimulation

**DOI:** 10.1186/1743-0003-7-27

**Published:** 2010-06-11

**Authors:** Jun Matsumoto, Toshiyuki Fujiwara, Osamu Takahashi, Meigen Liu, Akio Kimura, Junichi Ushiba

**Affiliations:** 1 School of Fundamental Science and Technology, Graduate School of Keio University, Kanagawa, Japan; 2 Department of Rehabilitation Medicine, Keio University School of Medicine, Shinjuku, Tokyo, Japan; 3 Clinical Laboratory, Ichikawa Rehabilitation Hospital, Chiba, Japan; 4 Keio University Tsukigase Rehabilitation Center, Shizuoka, Japan; 5 Department of Biosciences and Informatics, Faculty of Science and Technology, Keio University, Kanagawa, Japan

## Abstract

**Background:**

The mu event-related desynchronization (ERD) is supposed to reflect motor preparation and appear during motor imagery. The aim of this study is to examine the modulation of ERD with transcranial direct current stimulation (tDCS).

**Methods:**

Six healthy subjects were asked to imagine their right hand grasping something after receiving a visual cue. Electroencephalograms (EEGs) were recorded near the left M1. ERD of the mu rhythm (mu ERD) by right hand motor imagery was measured. tDCS (10 min, 1 mA) was used to modulate the cortical excitability of M1. Anodal, cathodal, and sham tDCS were tested in each subject with a randomized sequence on different days. Each condition was separated from the preceding one by more than 1 week in the same subject. Before and after tDCS, mu ERD was assessed. The motor thresholds (MT) of the left M1 were also measured with transcranial magnetic stimulation.

**Results:**

Mu ERD significantly increased after anodal stimulation, whereas it significantly decreased after cathodal stimulation. There was a significant correlation between mu ERD and MT.

**Conclusions:**

Opposing effects on mu ERD based on the orientation of the stimulation suggest that mu ERD is affected by cortical excitability.

## Background

Mu rhythm is a spontaneous characteristic feature of the electroencephalogram (EEG)/magnetoencephalogram (MEG) pattern that has 8-13 Hz activities that appear maximally over the central rolandic or sensorimotor area during a relaxed state. Mu rhythm is suggested to be present in 50-100% of healthy subjects [1], and is generally accepted as the idling rhythm engendered from the synchronized neurons involved in the thalamo-cortical loop [2,3].

The mu rhythm is attenuated by tactile stimulation, movement execution, and motor imagery, which are referred to as event-related desynchronization (ERD) [1,4,5]. Such ERD of mu rhythm, named mu ERD in this paper, are interpreted as the desynchronized activities of the activated neurons due to externally or internally paced events [2].

Amplitude changes due to externally or internally paced events are interpreted as the desynchronization or synchronization of neural activities of the cortex neurons. A recent study showed that mu ERD in preparation for contralateral extremity movement has some relationships with cortical activity seen on fMRI [6]. Several studies have shown that motor imagery of hand muscles increased the motor evoked potential (MEP) [7,8] and decreased the motor threshold (MT) of the contralateral primary motor cortex (M1) [9]. It is thought that there might be some relationship between cortical excitability and mu ERD.

Cortical excitability is modulated by transcranial direct current stimulation (tDCS). Anodal tDCS increases motor cortex excitability, whereas cathodal tDCS decreases it [10]. In this study, we studied whether tDCS application could modulate the cortical signal, such as mu ERD during right hand grasping images.

## Methods

### Subjects and experimental paradigms

Six healthy male subjects (age 30 ± 2 years, all right-handed) participated in this study after giving written informed consent. The investigation was planned in accordance with the Declaration of Helsinki, and was approved by the local ethical committee. No subject had a history of neurological disease or was receiving any acute or chronic medication affecting the central nervous system.

EEG signals were recorded from 20 Ag/AgCl disc electrodes (1 cm in diameter) with binaural reference according to the international 10-20 system of electrode placement (F1, Fz, F2, FC3, FC1, FCz, FC2, FC4, T3, C3, C1, Cz, C2, C4, T4, CP1, CPz, CP2, O1, O2) with the average of left and right earlobe reference to cover the motor areas of both hands and occipital area. Impedance for all channels was maintained below 10 kΩ through the experiment. All adjacent pairs of bipolar derivations of EEG were then used to check existence of mu ERD following motor imagery (see also 2.2. Quantification of ERD), and to determine the electrode pair showing the largest ERD. The selected bipolar EEG showing largest ERD was used for further analysis. Electromyogram (EMG) was simultaneously recorded from the first dorsal interosseous (FDI) with surface Ag/AgCl disc electrodes (1 cm in diameter) to confirm EMG activities during imagery tasks for avoiding unexpected muscle contraction. The electrodes were applied in belly-tendon recording. EEG and EMG were amplified, digitized (1000 Hz sampling frequency), and band-pass filtered (EEG 0.53-100 Hz, EMG 1.6-300 Hz) using a commercially available biosignal recorder (Neurofax EEG-9100, Nihon Kohden Corporation, Japan).

**Figure 1:**
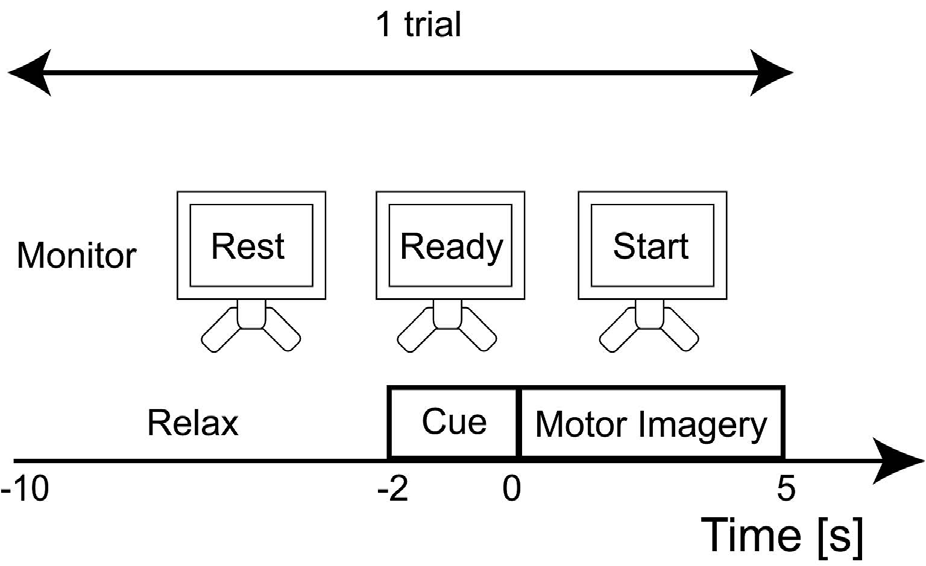
**The time course of a single trial consisted of three states: relaxed state, cue state, and motor imagery state**. The directions were displayed on a monitor in front of the subjects. A trial started with an 8-s period of a relaxed state during which the word "Rest" was shown at the center of the monitor. After that, a 2-s period during which a word "Ready" was shown began. Then, the word "Start" was presented for 5 s, and subjects were asked to imagine themselves grasping something with their right hand

Subjects sat in an armchair with their eyes open facing a computer monitor placed approximately 0.5 m in front of them at eye level. A trial started with an 8-s period of a relaxed state during which the word "Rest" was shown at the center of the monitor. After that, a 2-s period during which a word "Ready" was shown began. Then, the word "Start" was presented for 5 s, and subjects were asked to imagine themselves grasping a tennis-ball with their right hand [11]. The trial ended when the word "Rest" reappeared, and the next trial began after a break of 8 s (Fig. 1). Subjects were given no feedback about EEG changes to avoid a learning effect [12]. One session consisted of 20 trials, and three sessions were conducted before and after tDCS. There were breaks for about 5 min between sessions. All three sessions were completed within 30 minutes.

The tDCS was applied for 10 min through rectangular saline-soaked sponge electrodes (50 70 mm^2^) with a battery-driven stimulator (CX-6650, Rolf Schneider Electronics, Gleichen, Germany). The current intensity was set at 1 mA and the ramp time was set at 5 sec. The position of M1 was confirmed through the induction of the largest MEPs in the right FDI muscle with constant stimulus intensity using transcranial magnetic stimulation (TMS) with a figure-eight stimulation coil connected to a Magstim 200 magnetic stimulator (Magstim, Whitland, UK). One electrode was placed over the left M1 and the other was placed over the right supraorbital area. Three stimulation conditions (anodal, cathodal, and sham) were applied in each subject with a randomized sequence on different days to minimize carry-over effects. Each condition was separated from the preceding one by more than 1 week in the same subject. For anodal stimulation, the anodal electrode was placed over the left M1, and the cathodal electrode over the right supraorbital area. For cathodal stimulation, the electrodes were reversed; that is, the cathodal electrode was placed over the left M1 and the anodal electrode was placed over the right supraorbital area. For the sham stimulation, the current was applied for only 10 seconds to mimic the transient skin sensation at the beginning of actual tDCS without producing any conditioning effects on the brain [13]. For placing the stimulation electrode, three to four EEG electrodes over the stimulus site were removed after marking the scalp. After the tDCS stimulation, the EEG electrodes were set in same position as before. We here note that it took only less than 3 min for electrode replacement, and thus effect of elapsed time after tDCS on ERD measurement was limited.

Resting motor threshold (RMT) and active motor threshold (AMT) of the right FDI were measured before the placement of EEG electrodes for baseline EEG measurement. The threshold was determined with the FDI muscle at rest and during voluntary activity, and was defined as the minimum stimulus intensity that evoked a clearly identifiable EMG potential with a similar shape and latency in 5 of 10 successive stimuli [14]. For the measurement of RMT, the subject relaxed and EMG silence was monitored. RMT was defined as the lowest stimulus intensity capable of inducing MEPs greater than 50 μV in at least 5 of 10 trials. For determination of AMT, subjects made a steady contraction of about 5-10% of maximum, with the help of audiovisual feedback from the EMG.

### Quantification of ERD

Event-related trials of 5 s during motor imagery were selected for off-line data processing. All trials were visually assessed, and trials with artifacts (resulting from eye movement) as well as trials with increased EMG activity of the right FDI were excluded. All trials were segmented into successive 1-s windows with 100 overlapping samples, and the Fourier transformation with the Hanning window was applied in each segment. The power spectrum densities of each segment were estimated over the trials by Welch's averaged periodogram method [15].

The mu ERD was expressed as the percentage power decrease in relation to a 1-s reference interval before the direction of "Ready." The ERD was calculated for each time (resolution of 0.1 s) and frequency (resolution of 0.98 Hz) according to Equation (1).(1)

where A is the power spectrum density of the EEG at a certain frequency f [Hz] and time t [s] since imagery task was started, R is the power spectrum at the same frequency *f *[Hz] of the baseline period (a 1-s interval before the direction of "Ready" was displayed). The largest power decrease during motor imagery was selected as the value of mu ERD. Before tDCS application, the values of mu ERD were compared in all adjacent pairs of bipolar derivations of EEG, and determined the electrode pair showing largest value of mu ERD for individuals. Then, the values of mu ERD in three stimulation conditions (anodal, cathodal, and sham stimulation) were calculated from same bipolar derivation of EEG. All off-line analysis of EEG data was performed using MATLAB (The Mathworks Inc. USA).

### Statistics

A repeated measures two-way analysis of variance (ANOVA) was used to compare the mu ERD during imagery with main factors of type of stimulation (anodal, cathodal, and sham stimulation) and time (before and after stimulation). If ANOVA yielded a significant F value (p < 0.05), a post hoc test was carried out. One-way ANOVA was used to compare ERD before stimulation with type of stimulation (anodal, cathodal, and sham stimulation). Changes in ERD values were also assessed with a repeated measure ANOVA with the main factor being type of stimulation. If ANOVA yielded a significant F value (p < 0.05), a post hoc test was carried out.

To assess the relation between cortical excitability and the mu ERD, the Spearman rank correlation coefficient between the mu ERD value before simulation and MTs (RMT and AMT) was calculated. Statistical analysis was performed with SPSS 15.0J (SPSS Japan, Japan).

## Results

None of the subjects reported any adverse effects during or after the experiments. All subjects showed the mu ERD over the left cortex during motor imagery before tDCS. Three subjects showed the largest mu ERD at C3-FC3 and three showed the largest mu ERD at the electrode configuration adjacent to C3-FC3 (C1-FC1 for two subjects and C1-C3 for one subject). Further analysis was therefore performed with these electrode pairs to assess the effect of tDCS on mu ERD by motor imagery.

**Figure 2:**
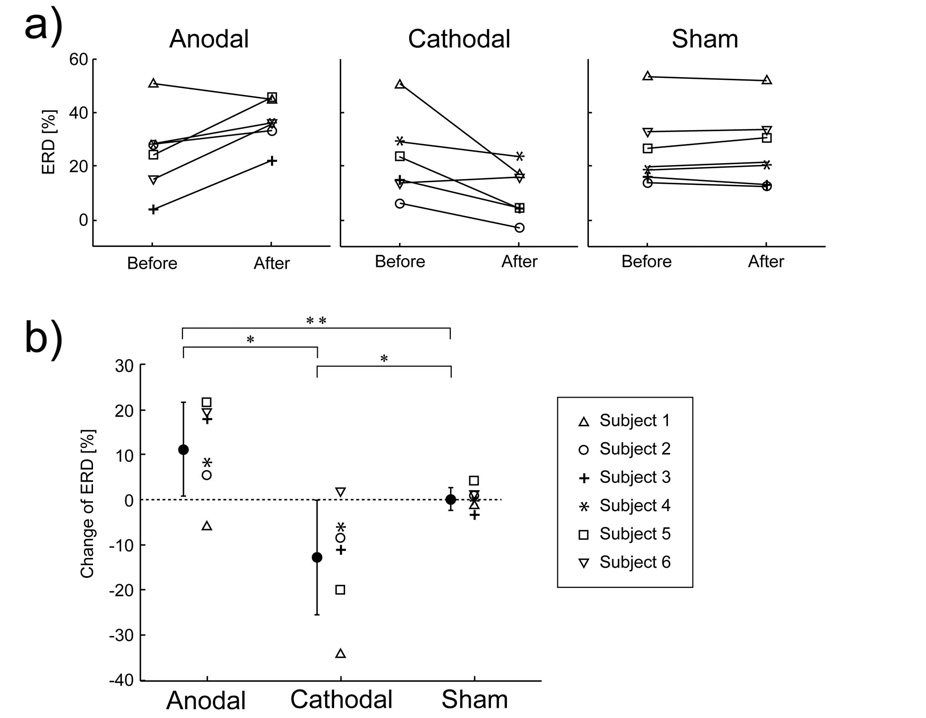
**Changes of mu ERD during the motor imagery of the right hand grasping something after the three types of tDCS (anodal, cathodal, and sham)**. a) mu ERD of each subject before and after the tDCS. Each symbol shows one subject. b) Changes of mu ERD before and after tDCS. The circles and vertical lines show the mean and standard deviation of the changes of mu ERD for each stimulation condition. *Post hoc LSD analysis showed a statistically significant difference (p < 0.05). ** Post hoc LSD analysis showed a statistically significant difference (p < 0.01).

In five of six subjects, the mu ERD was increased after anodal stimulation. Similarly, in five of six subjects, the mu ERD was decreased after cathodal stimulation (Fig. 2a). The mean (s.d.) change of the mu ERD value after anodal stimulation was 10.2% (10.0%), and the mean (s.d.) change of the ERD value after cathodal stimulation was -14.6% (12.1%). Differences between mu ERD values before and after tDCS are shown in Fig. 2b. A two-factor repeated measure ANOVA showed a significant interaction of stimulation and time (F2,10 = 17.47, p = 0.001). Post hoc paired t-test showed a significant increase of mu ERD after anodal tDCS (p = 0.047) and cathodal tDCS decreased mu ERD (p = 0.05). Repeated measure ANOVA showed the change of mu ERD values were significantly different among each stimulus (anodal, cathodal, and sham) (F2,10 = 17.47, p = 0.001). Post hoc LSD showed a significant difference between anodal-sham (p = 0.039), anodal-cathodal (p = 0.003) and cathodal-sham (p = 0.021) (Fig. 2b).

There was no significant change in the power spectrum of the resting state before and after each stimulus. Repeated measure ANOVA showed no significant difference among mu ERD before each stimulation (anodal, cathodal, and sham) (F2,10 = 0.39, p = 0.68).

**Figure 3:**
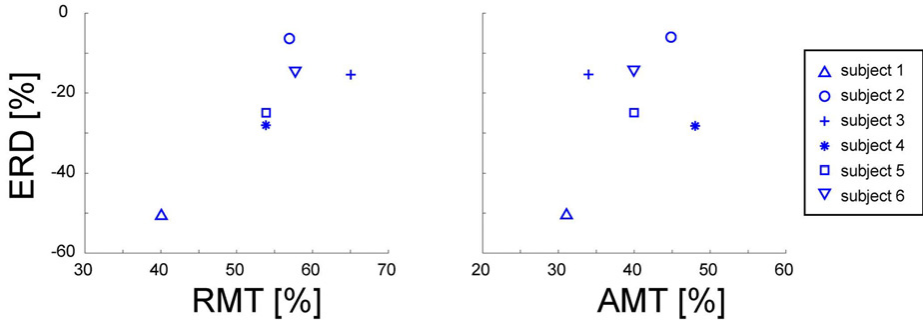
**Correlations between mu ERD and the MTs (RMT and AMT)**. Each symbol shows one subject. The x-axis shows the RMT (left) and AMT (right). The y-axis shows the mu ERD before the tDCS. Mu ERD showed a significant correlation with the RMT (*r *= 0.94, *p *< 0.05), whereas it did not show a significant correlation with AMT (*r *= 0.14)

The mean (s.d.) AMT was 40% (6%) and the mean (s.d) RMT was 55% (8%). There was a significant correlation between RMT and mu ERD of the session in which MT was determined (r = 0.94, p < 0.05), whereas there was no significant correlation between mu ERD and AMT (r = 0.14) (Fig. 3).

## Discussion

The mechanism of ERD is considered to be a decrease in synchrony of the underlying neuronal population [2].

Our data, further, showed that changes of cortical excitability induced by the tDCS influenced the mu ERD (i.e., increased ERD after anodal stimulation and decreased ERD after cathodal stimulation). Previous studies suggest that cortical excitability changes induced by the tDCS are due to modifications of membrane polarization [10,16,17] and synaptic mechanism [18]. Therefore changes of the mu ERD after the tDCS may be explained by changes in the oscillatory behavior of cortical neurons, such as membrane potentials in the primary motor area, and the probability of neurons firing according to input signals in response to motor imagery. Increased cortical excitability, such as depolarization of the membrane potential of the cortical neurons in the M1, will result in more activated and desynchronized neurons, based on the input signals from the motor imagery, which will increase mu ERD. Conversely, decreased cortical excitability, such as hyperpolarization of the membrane potential of cortical neurons, will lead to more deactivated and synchronized neurons, based on the input signals from the motor imagery, which will decrease mu ERD.

The ERD is suggested to be generated by the neural interconnection of the feedback loop involving the thalamo-cortical or cortico-cortical loop [2,19]. The tDCS seems to activate the intermediate neurons projecting to pyramidal tract neurons (PTN) in the cortex [18]. Therefore it is suggested that the mu ERD could be modulated by a change in excitability of the intermediate neurons projecting to the PTNs.

Our data show that the mu ERD is correlated with the RMTs of the M1. MT shows cortical excitability, or response to external input, of the most accessible part of the cortical area being investigated by TMS, because the intensity of the magnetic stimulus applied to assess MT is barely able to evoke a MEP and therefore does not spread to surrounding areas. The correlation between RMT and mu ERD suggests that mu ERD has some relationships with motor cortex excitability. However, we did not assess the motor cortex excitability after tDCS. And the number of subjects was limited. We need to further study to reveal the relationships between cortical excitability and mu ERD.

## Conclusions

Opposing effects on mu ERD based on the orientation of the stimulation suggest that mu ERD is affected by cortical excitability.

## Competing interests

No commercial party having a direct financial interest in the result of the research supporting this article has or will confer a benefit upon the authors or upon any organization with which the authors are associated.

## Authors' contributions

JM carried out the studies, analysis and interpretation of data, drafted the manuscript, and performed the statistical analysis. TF contributed to conception and design, carried out the studies, analysis and interpretation of data, and drafted the manuscript. OT carried out the studies, acquisition of data and analysis of data. ML and AK contributed to conception and design, and coordination and helped to draft the manuscript. JU contributed to conception and design, carried out the studies, analysis and interpretation of data and drafted the manuscript, and performed the statistical analysis. All authors read and approved the final manuscript.
